# Supplementary material for: Molecularly defined subpopulations of leptin receptor neurons dissociate the control of food intake from blood pressure
Source: bioRxiv. 2026 Mar 26:2026.03.26.714551. Preprint. [Version 1] doi: 10.64898/2026.03.26.714551 (PMC13041920; doi:10.64898/2026.03.26.714551)
Supplement: Supplement 1 [file NIHPP2026.03.26.714551v1-supplement-1.pdf]

bioRxiv preprint doi: https://doi.org/10.64898/2026.03.26.714551; this version posted March 26, 2026. The copyright holder for this preprint (which was not certified by peer review) is the author/funder, who has granted bioRxiv a license to display the preprint in perpetuity. It is made available under aCC-BY 4.0 International license.

Supplemental Figures and Legends

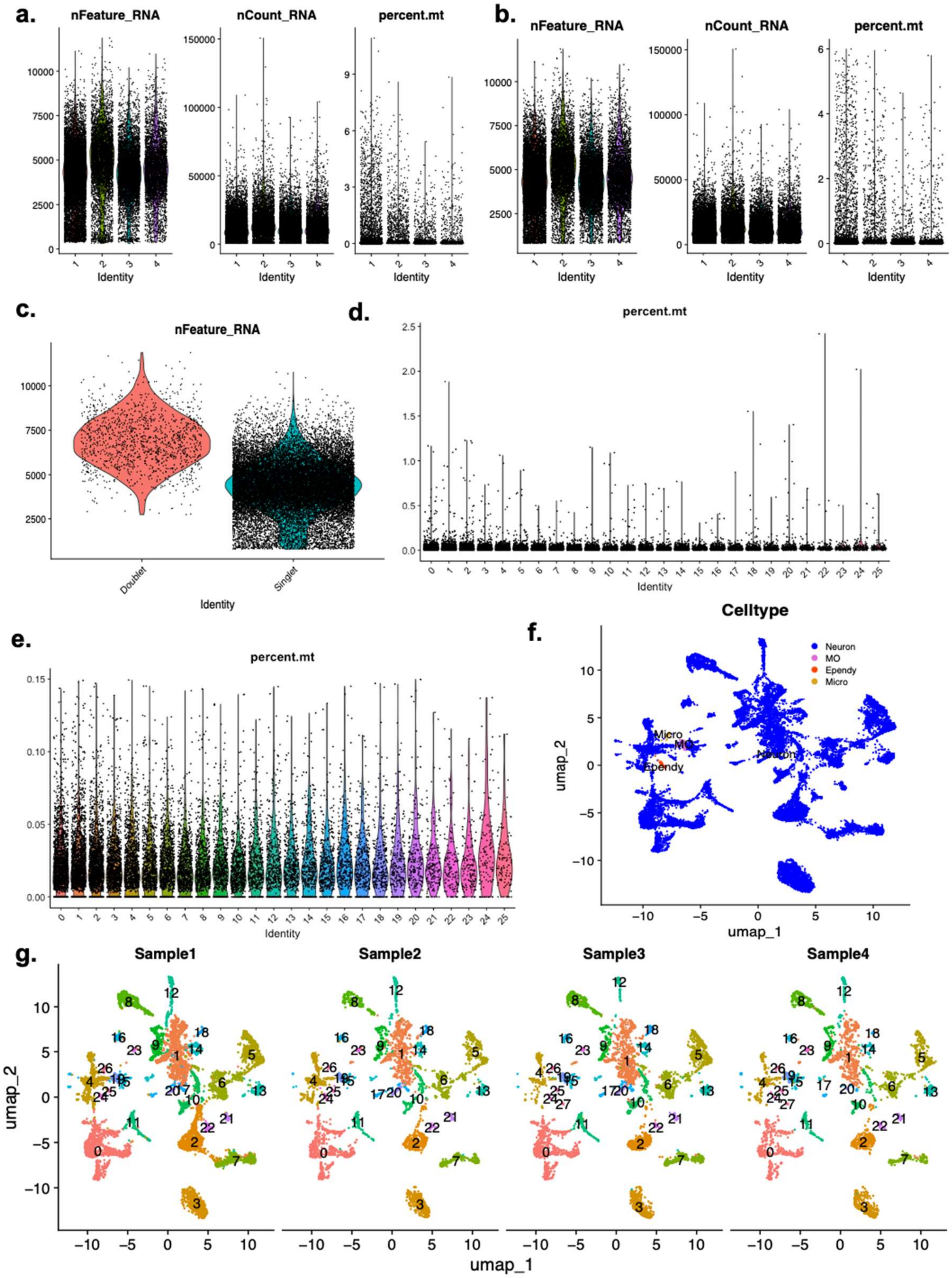

bioRxiv preprint doi: https://doi.org/10.64898/2026.03.26.714551; this version posted March 26, 2026. The copyright holder for this preprint (which was not certified by peer review) is the author/funder, who has granted bioRxiv a license to display the preprint in perpetuity. It is made available under aCC-BY 4.0 International license.

Supplemental Figure 1. Initial QC of LepRb$^{Sun1\text{-}GFP}$ mice sequencing dataset. (a) Distribution of raw features, feature counts and percent mitochondrial (mt) RNA across the four hypothalamic samples. (b) Distribution of features, feature counts and percent mt-RNA following the removal of all nuclei with fewer than 800 features and mtRNA greater than 6%. Feature number (c) for singlets and doublets. (d) Distribution of mtRNA between 0 and 2.5% across clusters defined by UMAP clustering of nuclei identified at singlets. (e) Distribution of mtRNA between 0 and 0.15%. (f) UMAP projection of nuclei colored by cell type and (g) UMAP projections of nuclei per sample following initial round of QC.

bioRxiv preprint doi: https://doi.org/10.64898/2026.03.26.714551; this version posted March 26, 2026. The copyright holder for this preprint (which was not certified by peer review) is the author/funder, who has granted bioRxiv a license to display the preprint in perpetuity. It is made available under aCC-BY 4.0 International license.

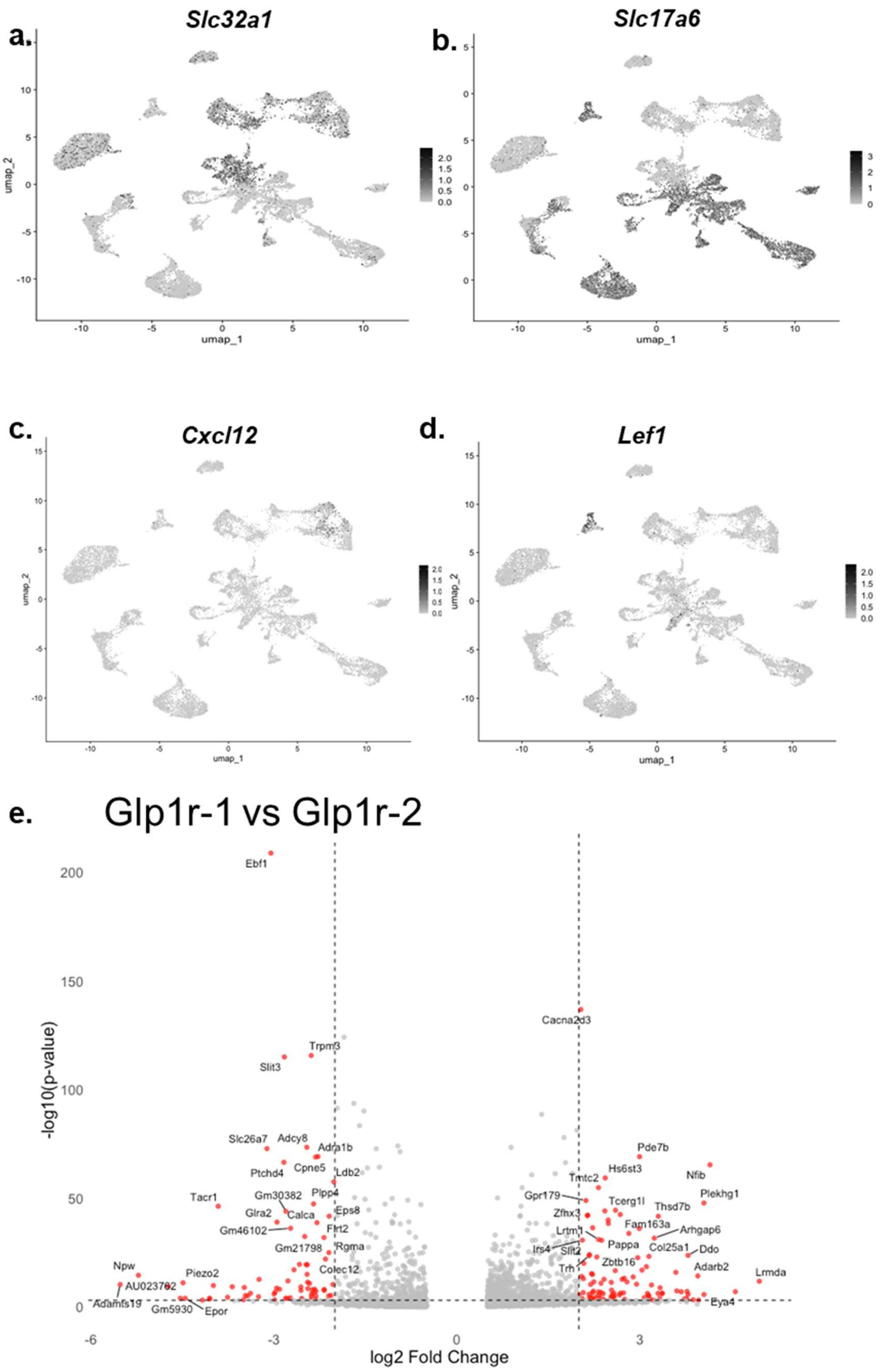

bioRxiv preprint doi: https://doi.org/10.64898/2026.03.26.714551; this version posted March 26, 2026. The copyright holder for this preprint (which was not certified by peer review) is the author/funder, who has granted bioRxiv a license to display the preprint in perpetuity. It is made available under aCC-BY 4.0 International license.

Supplemental Figure 2. Distribution of marker genes across all populations of *Lepr* neurons (a-d) and differential gene expression between Lepr$^{Glp1r\text{-}1}$ and Lepr$^{Glp1r\text{-}2}$ neuron populations. (a-d) Feature plots for *Slc32a1 (vGAT)* and *Slc17a6 (vGLUT2), Cxcl12, and Lef11* expression across neuronal nuclei post QC. (e) All genes differentially expressed between Lepr$^{Glp1r\text{-}1}$ and Lepr$^{Glp1r\text{-}2}$ clusters; p <0 .001 and $Log_2FC>2$ for genes indicated in red.

bioRxiv preprint doi: https://doi.org/10.64898/2026.03.26.714551; this version posted March 26, 2026. The copyright holder for this preprint (which was not certified by peer review) is the author/funder, who has granted bioRxiv a license to display the preprint in perpetuity. It is made available under aCC-BY 4.0 International license.

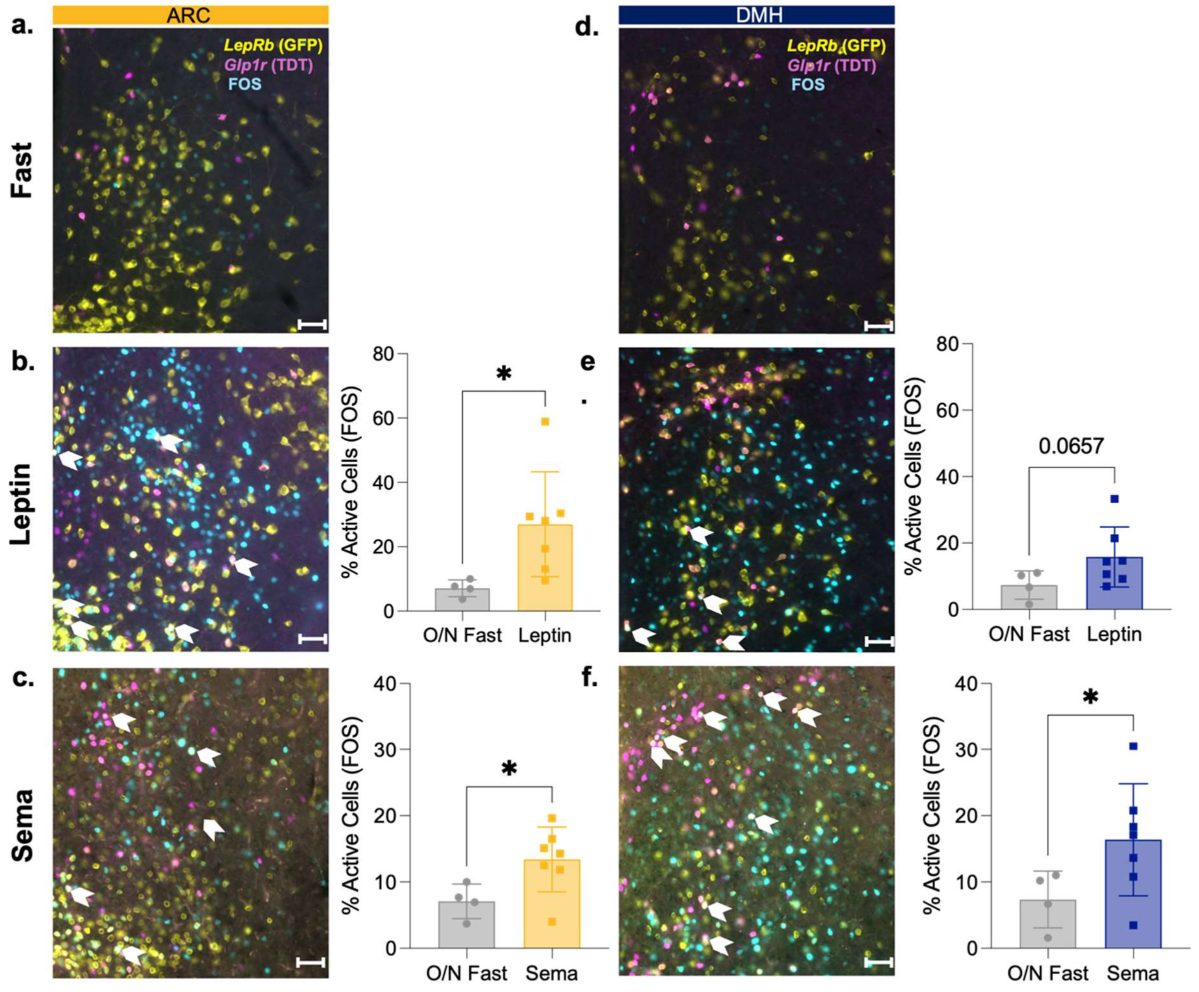

Supplemental Figure 3. FOS accumulation in response to leptin and semaglutide in ARC and DMH Lepr^{Glp1r} neurons. Shown are representative images of the ARC (a-c) and DMH (e-g) of Lepr^{GFP};Glp1r^{Tdt} mice showing *Lepr* (GFP; yellow) and *Glp1r* (tdTomato; pink) and FOS-immunoreactivity (blue) cells. Images show each area following a 14 hour fast alone (a, d, n=4 males), followed by leptin treatment (1 mg/kg, IP)(b, e; n= 2 females and 5 males) or semaglutide (Sema; 10 nmol/kg, IP)(c, f; n= 3 females and 4 males). White arrows indicate cells positive for all three signals. Scale bars= 50 um. Left panels show quantification of FOS colocalized with GFP+tdTomato

bioRxiv preprint doi: https://doi.org/10.64898/2026.03.26.714551; this version posted March 26, 2026. The copyright holder for this preprint (which was not certified by peer review) is the author/funder, who has granted bioRxiv a license to display the preprint in perpetuity. It is made available under aCC-BY 4.0 International license.

expressed as a percentage of total GFP+tdTomato cells. Mean +/-SEM are shown; *$p < 0.05$ by Welche's *t* test.

bioRxiv preprint doi: https://doi.org/10.64898/2026.03.26.714551; this version posted March 26, 2026. The copyright holder for this preprint (which was not certified by peer review) is the author/funder, who has granted bioRxiv a license to display the preprint in perpetuity. It is made available under aCC-BY 4.0 International license.

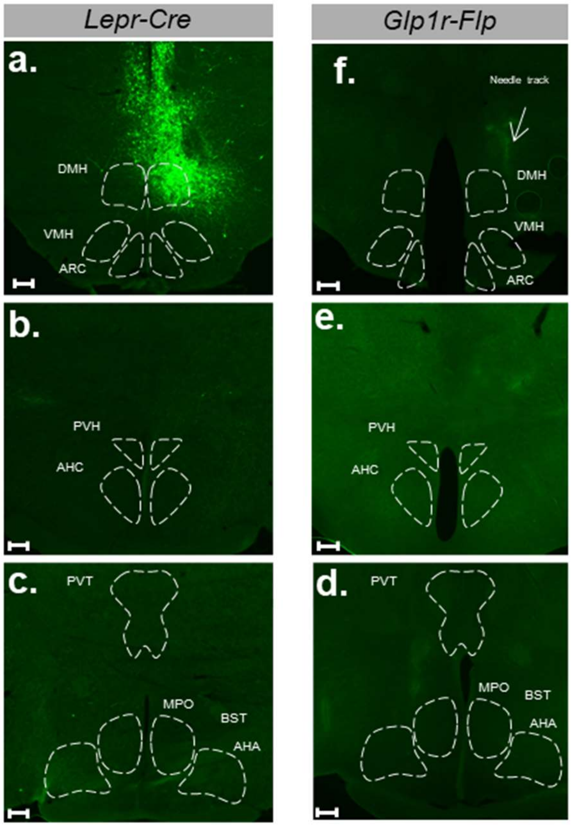

Supplemental Figure 4. Lack of retrograde tracing following injection of RADRR tracing system into *Lepr*^{Cre} only or *Glp1r*^{Flp} only animals. Shown are representative images showing GFP (green) cell bodies following the intra-DMH injection of the RADRR tracing system in *Lepr*^{Cre} mice (a-c; representative of n=2 males and 2 females) and *Glp1r*^{Flp} (d-f; representative of n=2 females). Needle track for the DMH of *Glp1r*^{Flp} mouse in (d) is indicated by a white arrowhead. Shown are the DMH injection site (a, d) and regions rostral to the DMH (b-c, e-f). Scale bar= 200 um.

bioRxiv preprint doi: https://doi.org/10.64898/2026.03.26.714551; this version posted March 26, 2026. The copyright holder for this preprint (which was not certified by peer review) is the author/funder, who has granted bioRxiv a license to display the preprint in perpetuity. It is made available under aCC-BY 4.0 International license.

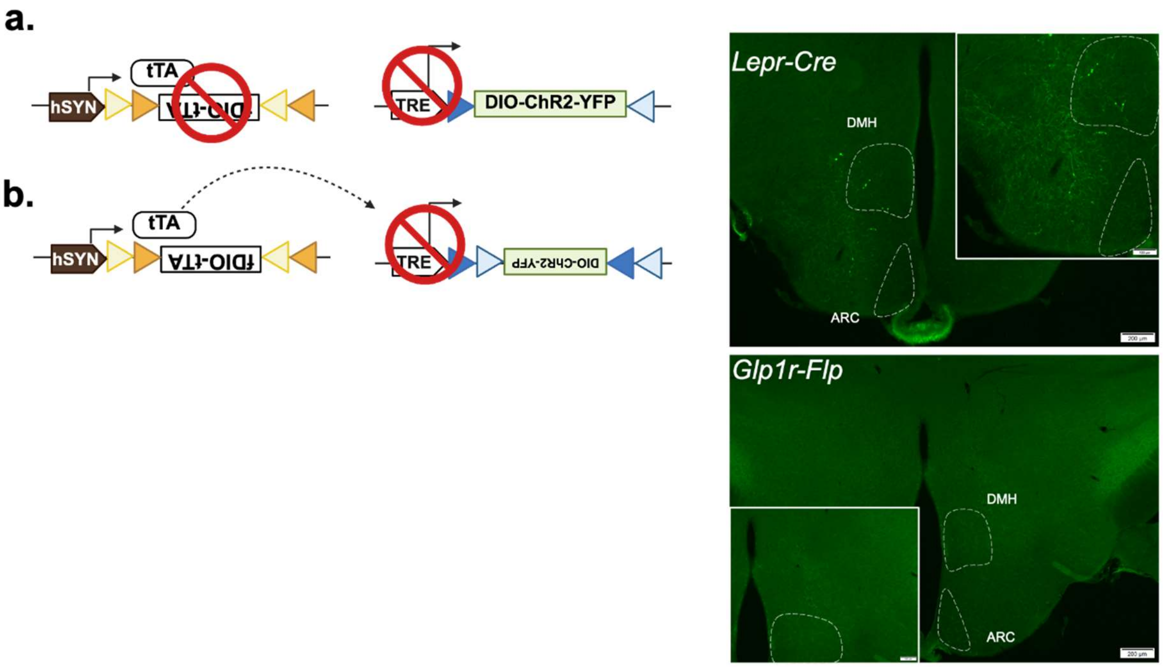

Supplemental Figure 5. Lack of detectable ChR2-YFP following injection of the tTARGIT system into *Lepr*^{Cre}- and *Glp1r*^{Flp}-only controls. Shown are (left panels) schematics of the result of injecting the indicated tTARGIT system plasmids into recombinase expressing cells in *Lepr*^{Cre} (a; representative of n=2 males and n=2 females) and *Glp1r*^{Flp} (b; representative of n=4 females) mice, along with a representative image of the ChR2-eYFP (GFP; green) detection in targeted area (DMH, right panels) in each control. Scale bar in main images= 200 um. Scale bar in digital zoom inset= image 100um.

bioRxiv preprint doi: https://doi.org/10.64898/2026.03.26.714551; this version posted March 26, 2026. The copyright holder for this preprint (which was not certified by peer review) is the author/funder, who has granted bioRxiv a license to display the preprint in perpetuity. It is made available under aCC-BY 4.0 International license.

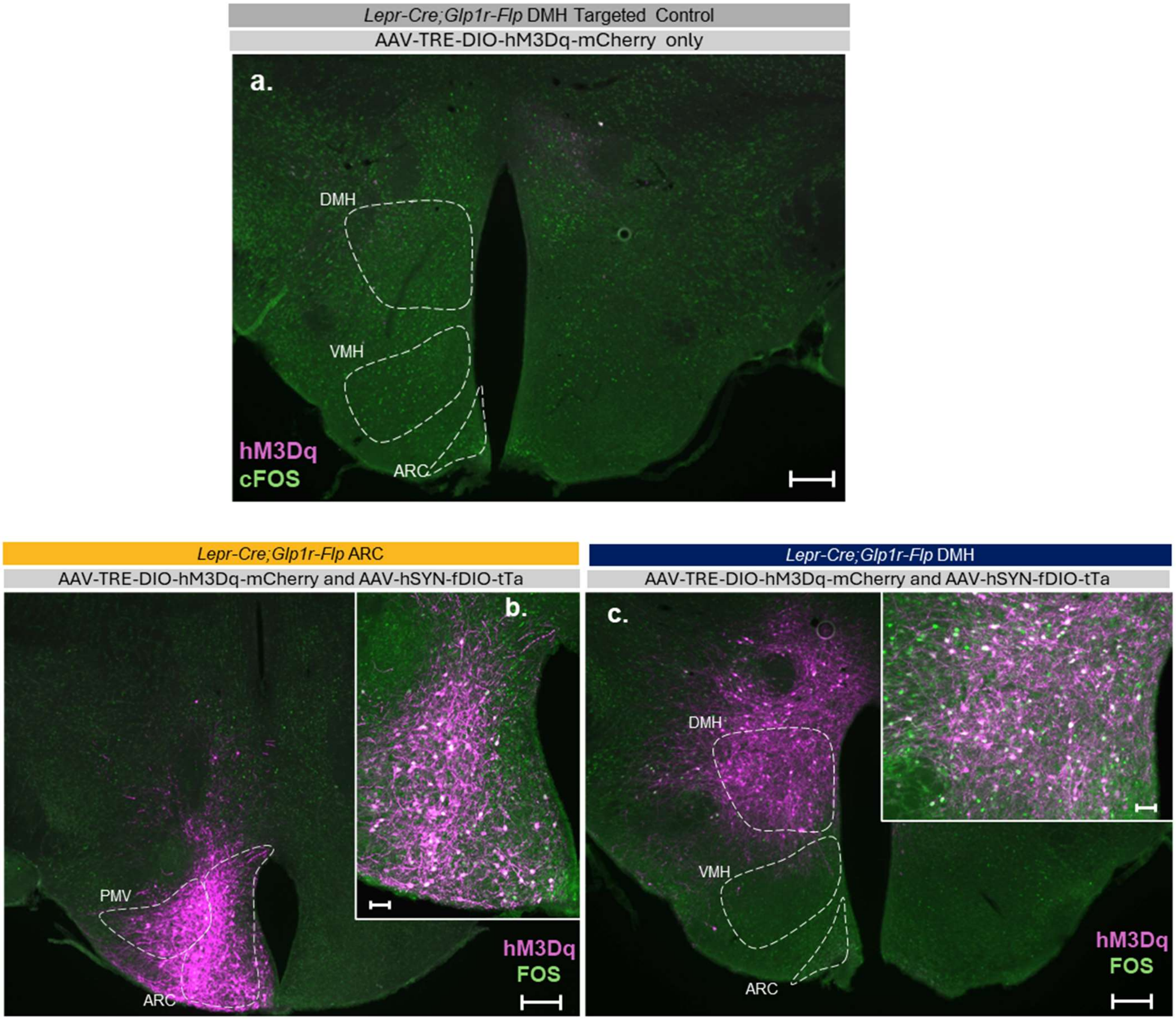

Supplemental Figure 6. Controls for tTARGIT hM3Dq expression and activation of ARC and DMH Lepr^{Glp1r} neurons in correctly targeted injections. Shown are representative images of mCherry (hM3Dq; magenta) and FOS-immunoreactivity (green) in the hypothalami of *Lepr^{Cre};Glp1r^{Flp}* mice injected into the DMH with AAV-TRE-DIO-hM3Dq-mCherry alone (top panel; representative of n=5 females and 3 males) or into the ARC (lower left; n=6 males, 5 females) or DMH (lower right, n=7 males, 4

bioRxiv preprint doi: https://doi.org/10.64898/2026.03.26.714551; this version posted March 26, 2026. The copyright holder for this preprint (which was not certified by peer review) is the author/funder, who has granted bioRxiv a license to display the preprint in perpetuity. It is made available under aCC-BY 4.0 International license.

females) with both AAV-TRE-DIO-hM3Dq-mCherry and AAV-hSYN-fDIO-tTa. Main panel scale bars= 200um, scale bars in digital zoom insets= 100um.

bioRxiv preprint doi: https://doi.org/10.64898/2026.03.26.714551; this version posted March 26, 2026. The copyright holder for this preprint (which was not certified by peer review) is the author/funder, who has granted bioRxiv a license to display the preprint in perpetuity. It is made available under aCC-BY 4.0 International license.

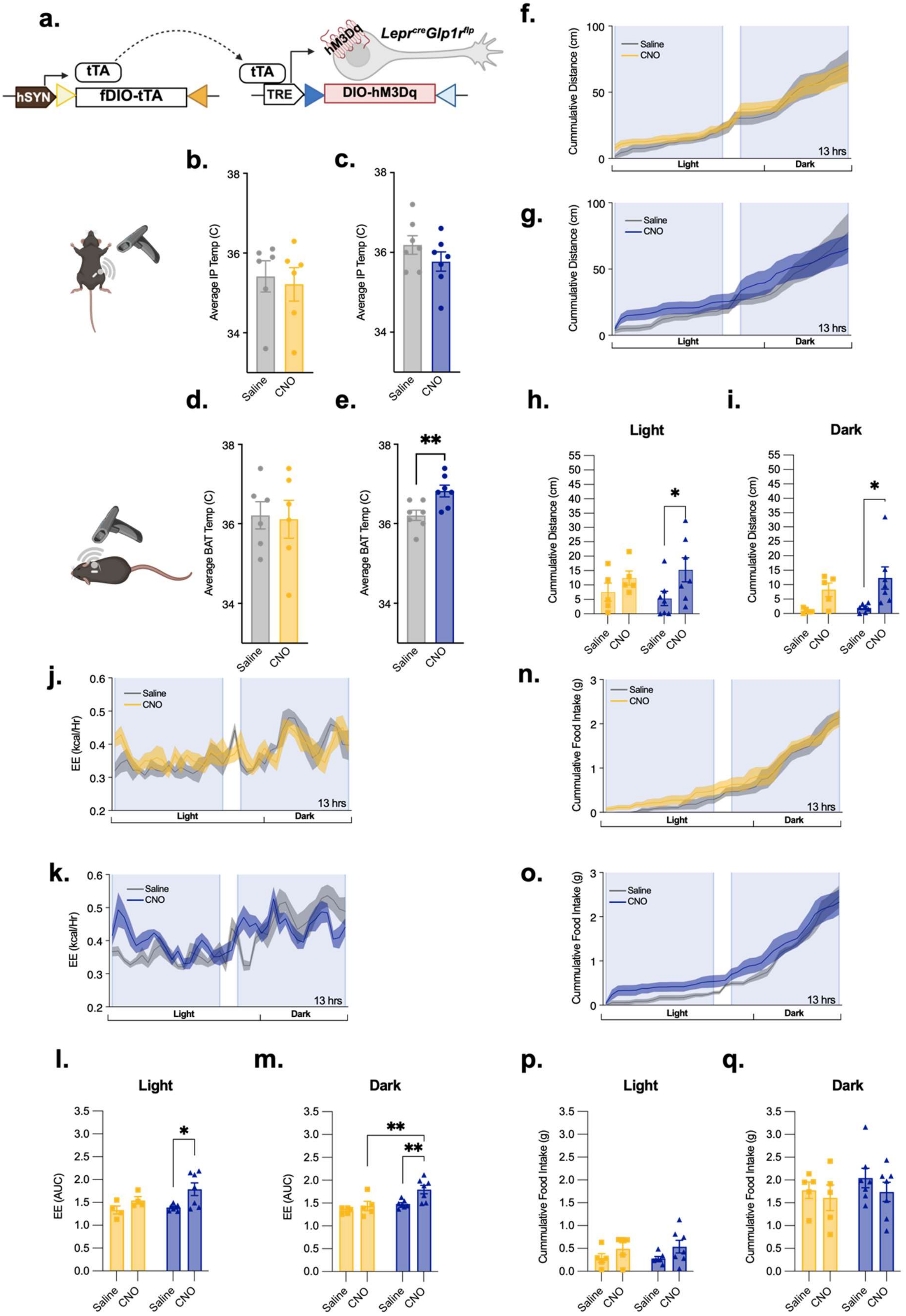

bioRxiv preprint doi: https://doi.org/10.64898/2026.03.26.714551; this version posted March 26, 2026. The copyright holder for this preprint (which was not certified by peer review) is the author/funder, who has granted bioRxiv a license to display the preprint in perpetuity. It is made available under aCC-BY 4.0 International license.

Supplemental Figure 7. Modulation of food intake and energy expenditure by ARC and DMH Lepr^{Glp1r} neurons in male mice. (a) Schematic of the tTARGIT viral system used to express hM3Dq. (b, c) Average change in IP (core body) temperature (b, c) or intrascapular (BAT) temperature (d, e) over the 360 minutes following the activation of ARC (b, d; n=5) or DMH (c, e; n=7) Lepr^{Glp1r} neurons. Shown are averages +/- SEM; $^{*}P < 0.05$ by Paired *t* test. (f-p) Continuous measurement of energy expenditure (f-i), cumulative locomotor activity (j-m), and cumulative food intake (n-q) following the injection of saline or CNO in Lepr$^{Glp1r\text{-}ARC\text{-}Dq}$ (yellow; n=6) or Lepr$^{Glp1r\text{-}DMH\text{-}Dq}$ mice (blue; n=7). (f, g, j, k, n, o) show continuous or cumulative measures of the indicated parameters over 13 hours; injections were performed at the beginning of the shaded areas. Graphs show summed values over the first hour (energy expenditure, locomotor activity) or six hours (food intake) following injection early in the light cycle (0900; h, l, p) or prior to the onset of the dark cycle (1600; i, m,q). Measurements were taken automatically using the SABLE metabolic cage system with 12 hour light and dark cycle. Comparisons were made between the third day of saline injections and first day of CNO injections. Bar graphs show averages +/- SEM; *p < 0.05, **p< 0.01 for the indicated comparisons by ANOVA with Fisher’s LSD post hoc test.

bioRxiv preprint doi: https://doi.org/10.64898/2026.03.26.714551; this version posted March 26, 2026. The copyright holder for this preprint (which was not certified by peer review) is the author/funder, who has granted bioRxiv a license to display the preprint in perpetuity. It is made available under aCC-BY 4.0 International license.

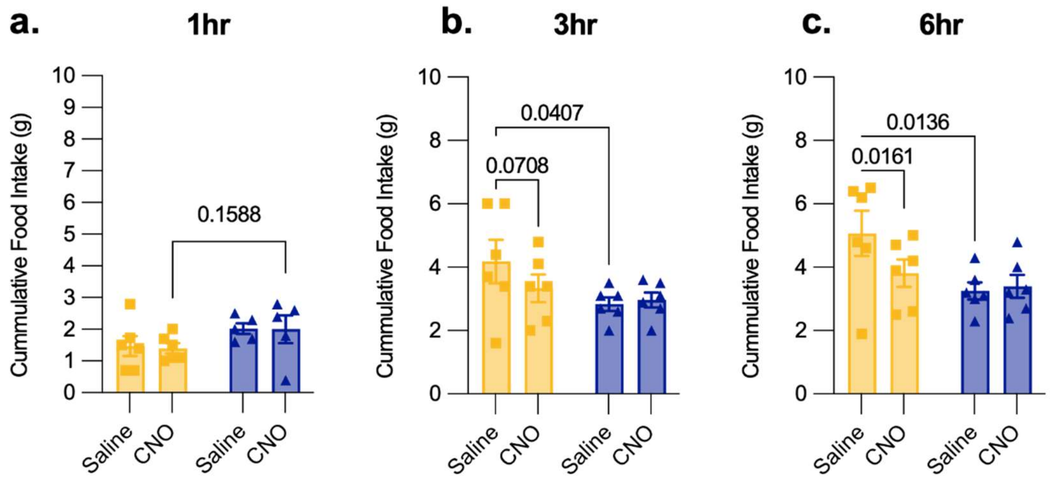

Supplemental Figure 8. Effects of activating ARC and DMH Lepr^{Glp1r} neurons on food intake during refeeding following an overnight fast. Cumulative food intake following refeeding and IP injection (b: ARC; n=3 male and 3 female, c: DMH; n=3 male and 3 female) at 1 hour (a), 3 hours (b) and 4 hours (c). Bar graphs show averages +/- SEM; indicated comparisons by ANOVA with Fisher's LSD post hoc test.
